# *Coleophora cytisicolella* sp. nov., a new species (Lepidoptera, Coleophoridae), from Austria and Hungary bred from *Chamaecytisus austriacus*

**DOI:** 10.3897/zookeys.1269.172969

**Published:** 2026-02-16

**Authors:** Attila Sándor Takács, Wolfgang Stark, Csaba Szabóky, Miklós Bozsó, Klaudia Kőszegi, Gábor Lendvai, Ignác Richter, Gábor Sramkó, Sándor Jordán

**Affiliations:** 1 Department of Evolutionary Zoology and Human Biology, University of Debrecen, H-4032 Debrecen, Egyetem tér 1, Hungary Department of Evolutionary Zoology and Human Biology, University of Debrecen Debrecen Hungary; 2 Government Office of Fejér County, Major Department of Agriculture Plant Protection and Soil Conservation Department, Ország út 23, H-2481 Velence, Hungary Institute of Plant Protection, University of Debrecen Debrecen Hungary; 3 Stockerauer Straße 16, A3430 Trübensee, Austria Department of Botany, University of Debrecen Debrecen Hungary; 4 Bécsi út 88, H1034 Budapest, Hungary National Food Chain Safety Office, Directorate of Food Chain Safety Laboratories Budapest Hungary; 5 Plant Health National Reference Laboratory, National Food Chain Safety Office, Directorate of Food Chain Safety Laboratories, Budaörsi út 141–145, H1118 Budapest, Hungary Government Office of Fejér County, Major Department of Agriculture Plant Protection and Soil Conservation Department Velence Hungary; 6 Faculty of the Agricultural and Food Sciences and Environmental Management, Institute of Plant Protection, University of Debrecen, Böszörményi Str. 138, 4032 Debrecen, Hungary Unaffiliated Trübensee Austria; 7 Tompa M. u. 38/C. H7000 Sárbogárd, Hungary Unaffiliated Budapest Hungary; 8 Malá Čausa, 289, SK97101 Malá Čausa, Slovakia Unaffiliated Sárbogárd Hungary; 9 Evolutionary Genomics Research Group, Department of Botany, University of Debrecen, H-4032 Debrecen, Egyetem tér 1, Hungary Unaffiliated Malá Čausa Slovakia; 10 HUN-REN–UD Conservation Biology Research Group, H-4032 Debrecen, Egyetem tér 1., Hungary HUN-REN–UD Conservation Biology Research Group Debrecen Hungary

**Keywords:** Austria, Carpathian Basin, Casebearer Moths, DNA barcode, Hungary, integrative taxonomy, new species

## Abstract

We describe *Coleophora cytisicolella***sp. nov**. (Lepidoptera: Coleophoridae), a new species from material collected in Austria and Hungary during recent fieldwork. The collected specimens were found only in these countries within the Pannonian Biogeographical Region, and were exclusively associated with *Chamaecytisus austriacus* (L.) Link (Fabaceae). The taxonomic status of the new species was determined by applying traditional macro- and micromorphological methods and genetic analysis, including genitalia examinations and DNA barcoding (cytochrome *c* oxidase subunit I). In addition to the results of morphological comparisons and genetic analysis, we present further information on the habitat, life history, and larval food plant of this species. Our results revealed that the examined individuals belong to a species new to science, which is a member of the *Coleophora genistae* Stainton, 1857 species group, and described here as *C. cytisicolella***sp. nov**. Based on the molecular results, the closest relative of the new taxon is *Coleophora bruttia*, a species described from southern Italy. Although the examined barcoding sequence poorly differentiated these taxa, the micromorphological features of the genitalia revealed their separate status.

## Introduction

The family Coleophoridae comprises more than 600 species in Europe (Huemer 2013; Rennwald and Rodeland 2024; Tóth et al. 2024). This is a little more than one-third of the estimated total of 1560 described species within the family (Baldizzone and Huemer 2024). Although the European *Coleophora* fauna is considered well documented (Emmet et al. 1996; Baldizzone et al. 2006; Tabell et al. 2024), the list of species continues to grow owing to ongoing fieldwork and surveys of museum and private collections.

Recent efforts to survey the taxonomic diversity within the family have yielded a substantial number of newly recorded species that have not been reported in Europe, or are even new to science. Species, such as *C. santonici* Baldizzone & Takács, 2022, *C. gazella* Toll, 1952, *C. avellanae* Tabell & Huemer, 2024, *C. gardesanella* Toll, 1953 (Stark and Buchner 2016; Baldizzone et al. 2022; Stark 2022, 2024; Tabell et al. 2024; Takács and Kőszegi 2024; Takács et al. 2024; Tóth et al. 2024) are only a few examples of *Coleophora* taxa recently described or found in Europe. Current research on the Coleophoridae is particularly intensive in the Pannonian Biogeographical Region (PBR), which covers Hungary and certain parts of neighbouring countries. Owing to its distinct biogeographical characteristics, this region is generally rich in species that are either endemic (e.g., *C. santonici*) or reach the westernmost boundary of their geographical range (e.g., *C. gazella*).

In this paper, we present another undescribed *Coleophora* species that was discovered in the PBR. In addition to a formal description of the new species, we provide morphological and genetic evidence to support our conclusion with additional information on its habitat, larval food plant, life history, and currently known geographical range.

## Material and methods

### Sample collection

The first specimens of an unknown *Coleophora* taxon were collected independently at two sites in the PBR. In the Vienna Basin at Oberweiden (Austria), Wolfgang Stark caught an unknown adult *Coleophora* on 30 April 2021, whereas at Kazal-hegy near Lovasberény (Hungary), A. Takács found 16 larval cases on *Chamaecytisus austriacus* (L.) Link (Fabaceae) on 13 September 2023.

During 2024, larval cases of the same species were found at nine new locations in Hungary: Fejér County, Sárbogárd (Bolondvár), and Székesfehérvár (Aszal-völgy); Pest County, Pilisborosjenő (Teve szikla), Pilisvörösvár (Kopár csárda), Kistarcsa (Küdői-hegy), and Isaszeg; Tolna County, Hőgyész (Lófej-hegy); Veszprém County, Nagyvázsony Veszprém, Látó-hegy (Table 1). Following A. Takács’s instructions, Wolfgang Stark also found the case in Austria during the autumn of 2024. The living material was collected or observed by B. Barabási, K. Kőszegi, G. Lendvai, Cs. Szabóky, M. Szilárd, T. Szalárdi, A. Takács and W. Stark.

**Table 1.** *Coleophora cytisicolella* sp. nov. specimens from Hungary and Austria used for molecular analyses.

Haplotype identification code	Specimen process ID in BOLD Database	BOLD BIN	Locality	Longitude, Latitude	Date of collection	Collector	Dev. stage	Sex
*C. cytisicolella* sp. nov. 1	COLHU025-24	BOLD:AGC5189	Lovasberény, Kazal-hegy Hungary	46°58'07"N, 18°33'07"E	16.04.2024	A. Takács	imago	male
*C. cytisicolella* sp. nov. 2	COLHU026-24	BOLD:AFA2793	Lovasberény, Kazal-hegy Hungary	46°58'07"N, 18°33'07"E	18.04.2024	A. Takács	imago	male
*C. cytisicolella* sp. nov. 3	COLHU027-25	BOLD:AGC5189	Lovasberény, Kazal-hegy Hungary	46°58'07"N, 18°33'07"E	16.04.2024	A. Takács	imago	male
*C. cytisicolella* sp. nov. 2	COLHU028-25	BOLD:AFA2793	Lovasberény, Kazal-hegy Hungary	46°58'07"N, 18°33'07"E	16.04.2024	A. Takács	imago	male
*C. cytisicolella* sp. nov. 2	COLHU029-25	BOLD:AFA2793	Lovasberény, Kazal-hegy Hungary	46°58'07"N, 18°33'07"E	16.04.2024	A. Takács	imago	male
*C. cytisicolella* sp. nov. 2	COLHU030-25	BOLD:AFA2793	Lovasberény, Kazal-hegy Hungary	46°58'07"N, 18°33'07"E	15.04.2024	A. Takács	imago	male
*C. cytisicolella* sp. nov. 4	NOELE2487-23	BOLD:AFA2793	Oberweiden, Austria	48°17'2.4"N, 16°49'58.8"E	30.04.2021.	W. Stark	imago	male
*C. cytisicolella* sp. nov. 5	COLHU031-25	BOLD:AFA2793	Lovasberény, Kazal-hegy Hungary	46°58'07"N, 18°33'07"E	21.07.2022	A. Takács	imago	male

The imagines and larval cases were collected exclusively from *Chamaecytisus austriacus*, a dwarf shrub native to the Eastern European steppe zone from East Austria to the Middle Volga region.

Adults were obtained by rearing larvae from the larval cases collected at Lovasberény (Table 1). The collected larval cases were placed on wild-collected *Chamaecytisus austriacus* plants, grown in A. Takács’s private garden, where the larvae overwintered. Each larval case was kept in a fine-mesh bag secured to the plants to prevent escape. The cases were placed in separate plastic vials a few days before the expected time of emergence, with suitable humidity maintained.

The cases, adults, and feeding signs of the larvae were photographed and described in detail. Pictures were taken with a Canon 450D camera (Canon Inc.) attached to a Carl Zeiss Stemi-2000 (Carl Zeiss Microscopy GmbH) binocular stereomicroscope and edited in Adobe Photoshop CS6 (Adobe Inc.). Eleven adults were sent for microscopic genitalia examination carried out by Ignác Richter (Slovakia). In the species description, the terminology in Baldizzone (2019) was used.

### Molecular study

Tissue samples from eight adults (one from Austria and seven from Hungary) were used to extract DNA and perform genetic analyses. Genomic DNA was isolated from one leg of each specimen using the E.Z.N.A.® Tissue DNA Kit (Omega Bio-tek, Inc.) following the manufacturer’s recommended protocol. Amplification of the cytochrome *c* oxidase I (COI) barcode region was performed with the primers LCO-1490 and HCO-2198 (Folmer et al. 1994). The PCR products were purified using the USB ExoSAP-IT® PCR Product Clean-Up reagent (Affymetrix), and the amplicons were sequenced with the LCO-1490 primer (BaseClear B.V., Netherlands).

Using the BOLD System’s Identification Request function, 11 haplotypes of the four most closely related *Coleophora* species (*C. bruttia*Baldizzone 2023, *C. genistae* Stainton, 1857, *C. saturatella* Stainton, 1850, *C. trifariella* Zeller, 1849) were collected and used in a phylogenetic analysis. Sequence alignment and calculation of pairwise genetic distances were conducted using MEGA 7 (Kumar et al. 2016). The number of analysed haplotype sequences of *C. cytisicolella* sp. nov. was reduced to represent only the unique haplotypes; i.e., each haplotype was represented by a single sequence, regardless of the number of specimens observed in the corresponding haplotype. Alignment of the downloaded haplotype sequences was performed using ClustalW (Larkin et al. 2007), with default parameters. The most appropriate nucleotide substitution model was determined using the Bayesian Information Criterion (BIC). The Tamura-3-parameter model with Gamma Distribution (T92+G) (Tamura 1992) was selected for distance analysis and phylogenetic reconstruction. The phylogenetic tree was constructed using the Minimum Evolution (ME) method implemented in MEGA 7 with the default initial rearrangement settings, and was rooted to *Coleophora niveicostella* (BOLD Sample ID: ABOLA798-15). A bootstrap analysis using 1000 replicates was performed to estimate the support for each node.

### Abbreviations

**AT** Attila Sándor Takács, Velence, Hungary

**BB** Brigitta Barabási

**CsSz** Csaba Szabóky, Budapest, Hungary

**GB** Giorgio Baldizzone, Asti, Italy

**GL** Gábor Lendvai, Sárbogárd, Hungary

**GR** Gerhard Rotheneder, Siebenhirten bei Böheimkirchen, Austria

**HNHM** Hungarian National Museum Public Collection Centre – Hungarian Natural History Museum, Budapest

**IgR** Ignác Richter, Malá Čausa, Slovakia

**JT** Jukka Tabell, Hartola, Finland

**KK** Klaudia Kőszegi, Budapest, Hungary

**MB** Miklós Bozsó, Budapest, Hungary

**PB** Peter Buchner, Schwarzau am Steinfeld, Austria

**PBR** Pannonian Biogeographical Region

**SJ** Sándor Jordán, Debrecen, Hungary

**SzM** Szilárd Molnár

**TSz** Tímea Szalárdi

**WS** Wolfgang Stark, Trübensee, Austria

**Gen. slide** genitalia dissection

**In coll**. in collection

## Results

### Description of the new species

A total of 99 (47 males, 52 females) specimens were collected, the data of which are listed here.

#### Coleophora cytisicolella

Taxon classificationAnimaliaLepidopteraColeophoridae

Takács, Stark, Szabóky & Bozsó sp. nov.

A806E120-BFCD-525F-8022-67230330A132

https://zoobank.org/6D61541B-A716-45DF-8680-F8AD2346C8D2

Figs 1 A, B, 2, 3

##### Type material.

***Holotype***: ♂; Hungary • Fejér County; Lovasberény, Kazal-hegy, 47°17'51"N, 18°33'53"E; 240 m; 15 April 2024, ex larva on *Chamaecytisus austriacus*, gen. slide IgR 35384; leg. AT, in coll. HNHM. BOLD Sample ID: COLHU030-25. The genitalia were mounted on a slide in Euparal, in coll. HNHM. ***Paratypes***: Hungary • same location, same host plant as the holotype, but: 1♂; gen. slide IgR, 34836; 21 July 2022; UV led light trap; leg. AT, in coll. HNHM; 1♂; gen. slide IgR, 36184 (as *Coleophora genistae* Stainton, 1857); 08 August 2023; UV led light trap; leg. AT, in coll. HNHM; • same location, same host plant as the holotype, but: 2♂♂; gen. slide IgR, 35379; (BOLD Sample ID: COLHU026-24), gen. slide IgR, 35196, 15 April 2024; leg. AT, in coll. AT; • same location, same host plant as the holotype, but: 2♀; gen. slide IgR 35385; (BOLD Sample ID: COLHU029-25), gen. slide IgR 35195; 16 April 2024; leg. AT, in coll. AT & IR; • same location, same host plant as the holotype, but: 6♂♂; gen. slide IgR: 35380; gen. slide IgR: 35381; (BOLD Sample ID: COLHU028-25); gen. slide IgR: 35383; 35195; (BOLD Sample ID: COLHU025-24), gen. slide IgR: 35382; 16 April 2024; leg. AT, in coll. AT & IR; • same location, same host plant as the holotype, but: 2♂♂, 2♀♀; 24 March 2025; leg. AT, in coll. AT; • same location, same host plant as the holotype, but: 1♀; 26 March 2025; leg. AT, in coll. CsSz; • same location, same host plant as the holotype, but: 1♂; 28 March 2025; leg. AT, in coll. GB; • same location, same host plant as the holotype, but: 1♀; 29 March 2025; leg. AT, in coll. AT; • same location, same host plant as the holotype, but: 1♀; 30 March 2025; leg. AT, in coll. GB; • same location, same host plant as the holotype, but: 1♂; 31 March 2025; leg. AT, in coll. AT; • same location, same host plant as the holotype but: 1♂, 1♀; 11 April 2025; leg. AT & KK, in coll. AT; • same location, same host plant as the holotype, but: 2♀♀; 12 April 2024; leg. AT & KK, in coll. AT; • same location, same host plant as the holotype, but: 1♂; 14 April 2025; leg. AT & KK, in coll. AT; 1♀; 19 April 2025; leg. AT & KK, in coll. AT; • same location, same host plant as the holotype, but: 1♂; 29 April 2025; leg. AT & KK, in coll. AT; • same location, same host plant as the holotype, but: 1♀; 12 May 2025; gen. slide IgR 36392; leg. AT & KK, in coll. AT; • same location, same host plant as the holotype, but: 1♀; 13 May 2025; leg. AT & KK leg., in coll. AT; • same location, same host plant as the holotype, but: 1♀; 21 May 2025; leg. AT & KK leg., in coll. AT; 2♀♀; Hungary Fejér County, Sárbogárd, Bolondvár; 46°54'29.0"N, 18°39'48.7"E; 25 March 2025; ex larva on *Chamaecytisus austriacus*, leg. AT & GL, in coll. AT; • same location, same host plant, but: 2♂♂; 31 March 2025; leg. AT & GL, in coll. GB & CsSz; 2♂♂, 3♀♀; Hungary • Fejér County, Székesfehérvár; Aszal-völgy; 47°14'33.1"N, 18°25'36.1"E; 65 m, 31 April 2025; ex larva on *Chamaecytisus austriacus*, leg. AT & KK, in coll. AT, IR (1♂) & CsSz (1♀); • same location, same host plant, but: 2♂, 3♀; 09 April 2025; leg. AT & KK, in coll. AT; 2♀♀; Hungary • Pest County, Isaszeg; Szarka berek; 47°32'15.6"N, 19°22'01.3"E, 250 m; 06 April 2025; ex larva on *Chamaecytisus austriacus*, leg. AT, in coll. AT; • same location, same host plant, but: 2♂♂; 07 April 2025; leg. AT, in coll. AT & GB; • same location, same host plant, but: 1♀; 09 April 2025; leg. AT, in coll. AT; 1♀; Hungary • Pest County, Kistarcsa; Küdői-hegy; 295 m; 26 March 2025; 47°32'00.6"N, 19°19'35.7"E; ex larva on *Chamaecytisus austriacus*, leg. AT, in coll. CsSz; • same location, same host plant, but: 1♂, 1♀; 31 March 2025; leg. AT, in coll. CsSz; • same location, same host plant, but: 1♂, 1♀; 11 April 2025; leg. AT, in coll. AT & JT (1♂); • same location, same host plant, but: 2♀♀, 12 April 2025; leg. AT, in coll. AT & IR; • same location, same host plant, but: 2♀♀; 13 April 2025; leg. AT, in coll. AT; 1♂; Hungary • Pest County, Pilisborosjenő; Teve-szikla; 300 m; 26 March 2015; 47°36'50.5"N, 18°58'40.2"E; ex larva on *Chamaecytisus austriacus*, leg. CsSz, in coll. CsSz; • same location, same host plant, but: 1♀; 3 April 2025; leg. CsSz, in coll. AT; • same location, same host plant, but: 1♂, 3♀; 05 April 2025; leg. CsSz, in coll. AT, GB (♀) & JT (♀); • same location, same host plant, but: 1♂; 04 April 2025; leg. CsSz, in coll. AT 1♂; Hungary • Pest County, Pilisvörösvár, Kopár csárda; 250 m; 26 March 2025; 47°62'20"N, 18°86'64"E; ex larva on *Chamaecytisus austriacus*, leg. AT & CsSz, in coll. CsSz; • same location, same host plant, but: 1♂; 31 March 2025; leg. AT & CsSz, in coll. CsSz; • same location, same host plant, but: 1♂; 15 April 2025; leg. AT & CsSz, in coll. AT; • same location, same host plant, but: 1♂; 16 April 2025; leg. AT & CsSz, in coll. AT; • same location, same host plant, but: 1♂; 17 April 2025; leg. AT & CsSz, in coll. AT; 1♀; Hungary • Tolna County, Hőgyész; Lófej-hegy; 200 m; 02 April 2025; 46°46'95"N, 18°44'09"E; ex larva on *Chamaecytisus austriacus*, leg. AT & GL, in coll. AT; • same location, same host plant, but: 2 ♂♂, 1♀; 09 April 2025; leg. AT & GL, in coll. AT; 1♀; Hungary • Veszprém County, Nagyvázsony; 26 March 2025; 46°54'33.1"N, 18°39'51.0"E; ex larva on *Chamaecytisus austriacus*, leg. AT, BB & TSz, in coll. CsSz; • same location, same host plant, but: 1♀; 03 April 2025; leg. AT, BB, & TSz, in coll. AT; • same location, same host plant, but: 1♀; 05 April 2025; leg. AT, BB & TSz, in coll. GB; • same location, same host plant, but: 1♂; 11 April 2025; leg. AT, BB, & TSz, in coll. AT; • same location, same host plant, but: 1♀; 13 April 2025; leg. AT, BB, & TSz, in coll. AT; 2♂♂; Hungary • Veszprém County, Veszprém; Látó-hegy; 03 April 2025; 47°05'17.5"N, 17°56'30.7"E; 220 m; ex larva on *Chamaecytisus austriacus*, leg. AT, SzM, & TSz, in coll. AT; • same location, same host plant, but: 1♂, 2♀; 04 April 2024; leg. AT, SzM & TSz, in coll. AT; • same location, same host plant, but: 1♂, 1♀; 05 April 2025; leg. AT, SzM. & TSz, in coll. AT; • same location, same host plant, but: 1♀; 21 May 2025; leg. AT, SzM & TSz, in coll. AT. 1♂; Austria, Oberweiden, 48°17'03"N, 16°49'60"E; 30 April 2021, WS leg., in coll. WS. BOLD Sample ID: NOELE2487-23; • same location, host plant *Chamaecytisus austriacus*, 3 October 2024, 1♀ Austria, Oberweiden, 16 May 2025, WS, leg., in coll. WS, same location, same host plant and date, but 1♂ and 1♀; Austria, Oberweiden, 17 May 2025, WS leg., in coll. WS, same location, same host plant and date, but 1♂; Austria, Oberweiden, 18 May 2025, WS leg., in coll. WS, same location, same host plant and date, but 2♀♀; Austria, Oberweiden, 19 May 2025, WS leg., all 7 specimens in coll. WS.

**Figure 1.**
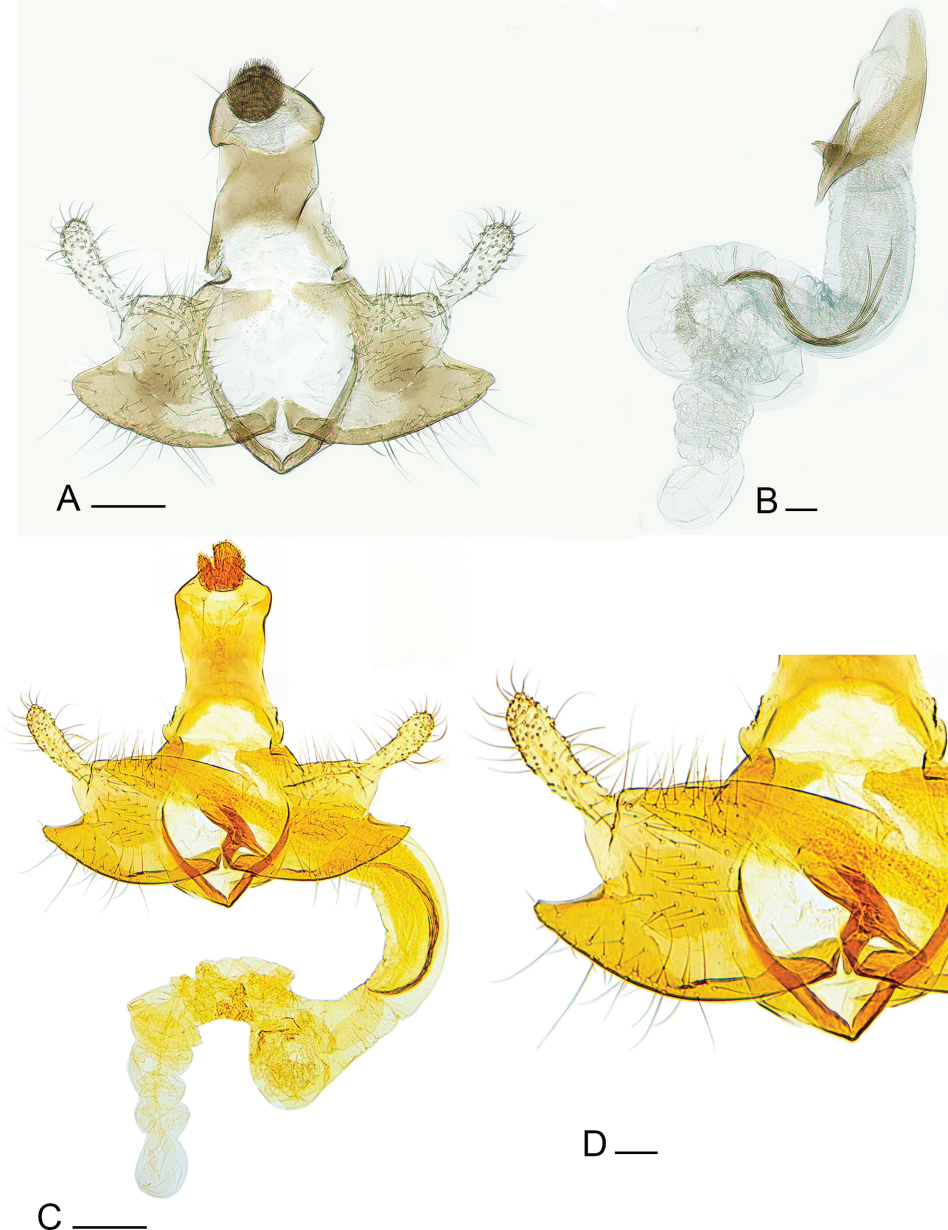
Genitalia of *Coleophora cytisicolella* sp. nov. **A**. Male genitalia of *C. cytisicolella* sp. nov., gen. slide IgR 35384, holotype, Fejér County, Lovasberény, loess wall, ex. larva on *Chamaecytisus austriacus*, 16 April 2024, leg. AT; **B**. Phallotheca of *C. cytisicolella* sp. nov. holotype, Fejér County, Lovasberény, loess wall, ex. larva on *Chamaecytisus austriacus*, 16 April 2024, leg AT; **C**. Male genitalia of *C. bruttia*Baldizzone 2023, holotype, gen. slide Bldz 16202; **D**. Enlarged detail of valva and phallotheca of *C. bruttia*Baldizzone 2023. Photo: GB, IR. Scale bars: 0.2 mm.

##### Diagnosis.

Adult males differ from *C. bruttia* in the following characters: forewings are overall darker with pattern of two white longitudinal strips dividing the forewing into three sections. Forewing is yellowish-brown at base, gradually turning into warm chestnut brown in the middle. The costal margin is broad, white from the base to almost the wingtip. The head is pale yellow at the central part.

**Figure 2.**
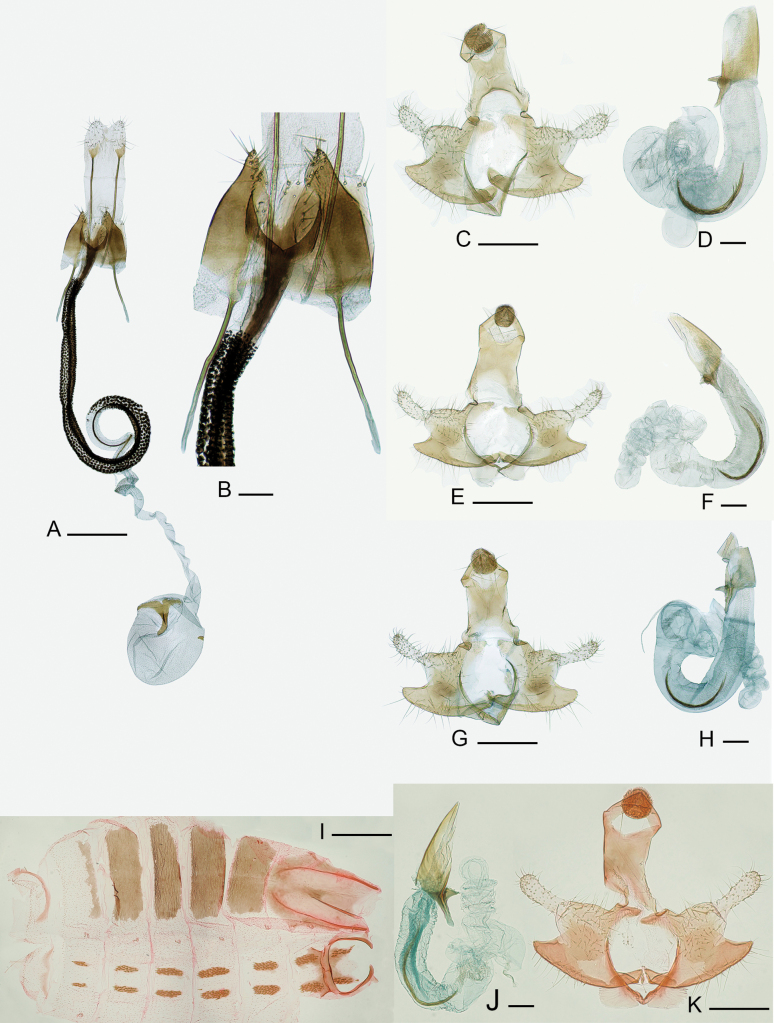
Genitalia of *Coleophora cytisicolella* sp. nov. **A**. Female genitalia of *C. cytisicolella* sp. nov., gen. slide IgR 35195, holotype, Fejér County, Lovasberény, loess wall, ex. larva on *Chamaecytisus austriacus*, 16 April 2024, leg. AT; **B**. Part between apophyses and ductus bursae enlarged; **C–H**. Male genitalia of *C. cytisicolella* sp. nov, paratypes, gen. slide IgR 35382; 35385; 36184; **I–K**. Male genitalia and abdomen of *C. cytisicolella* sp. nov., gen. slide PB 35195 holotype, Austria, Oberweiden, 30 April 2021, leg. WS. Photos: IgR and PB. Scale bars: 0.4 mm.

The male genitalia (Figs 1A, 1B, 2C–K) exhibited significant similarities to those of *C. bruttia* (Baldizzone 2023) (Fig. 1C, D) with some notable differences: the gnathos is larger, broader, and more rounded, densely spinose, and the basal arm of the gnathos is rounded. The tegumen is somewhat longer and slightly broader. The pedunculus is broader and more sclerotised than that of *C. bruttia*. The valvula is somewhat wider and more or less angular in shape. The arm of the cucullus is broader and more bulbous towards the apex. The sacculus is longer and more rounded, tapering at the base. The vinculum is U-shaped. In the phallotheca, the cornutus forms a looser, more sclerotised bundle that is spreading at the apex.

Based on the habitus of the adult specimens and the structures of the male genitalia, *Coleophora cytisicolella* belongs to the *C. genistae* (Stainton, 1857) species group (Baldizzone 2019; Tabell et al. 2024).

The male genitalia structure of *Coleophora cytisicolella* sp. nov. also shows similarities to the following species: *C. trifariella* Zeller, 1849, *C. genistae* Stainton, 1857, and *C. saturatella* Stainton, 1850. In *C. trifariella* and *C. genistae*, the gnathos is rounded but less spinose, whereas the gnathos of *C. saturatella* is oval in shape. The arm of the cucullus in *C. trifariella* and *C. genistae* is uniformly wide towards the apex and only slightly hairy; in *C. trifariella*, the cucullus is oriented nearly horizontally, and in both species, the costa is straight, lacking any bulge. The cucullus of *C. saturatella* is more slender than that of *C. cytisicolella*, with slightly convex costal part in the middle section of it. The sacculus is rounded in *C. trifariella* and *C. genistae*, while it is pointed in *C. saturatella*. In all three species, the cornutus is sclerotised and forms a loose bundle (Tabell et al. 2024).

The female genitalia structure of *Coleophora cytisicolella* sp. nov. (Fig. 2A, B) shows similarities to the following species: *C. trifariella* Zeller, 1849, *C. genistae* Stainton, 1857, and *C. saturatella* Stainton, 1850. In *C. trifariella* and *C. genistae*, the papillae anales are trapezoidal, and the anterior apophyses are short. In *C. saturatella*, the anterior apophyses are also short, but the papillae anales protrude from the tergum. The ostium in all three species is pointed and V-shaped, whereas it is rounded and U-shaped in *Coleophora cytisicolella* sp. nov. In *C. trifariella* and *C. genistae*, the basal plate of the signum is heavily sclerotised and C-shaped, while the basal plate of *C. saturatella* is shaped like a lying ‘B’ (Tabell et al. 2024).

##### Description.

**Figure 3.**
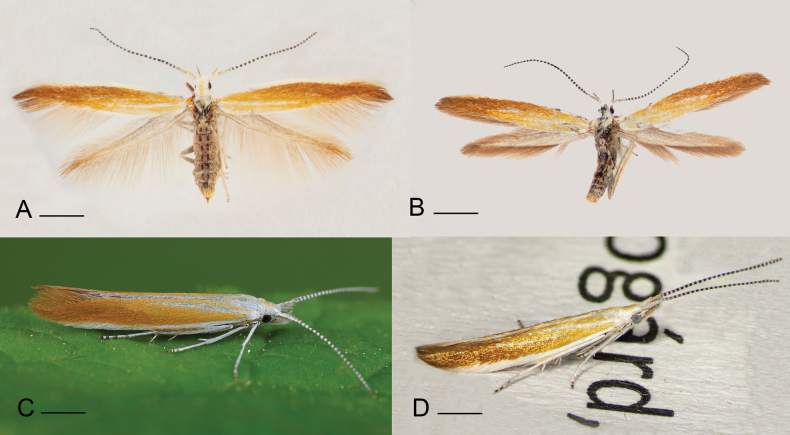
Adults of *Coleophora cytisicolella* sp. nov. **A**. Female, paratype, Fejér County, Lovasberény, Kazal-hegy, 16 April 2024, gen. slide IgR 35385, leg. AT; **B**. Male, holotype, Fejér County, Lovasberény, loess wall 16 April 2024, gen. slide IgR 35384; **C**. Male, Oberweiden, Austria, 17 May 2025, leg. WS; **D**. Female, Veszprém County, Veszprém, Látó-hegy, 18 April 2025, leg. AT. Photos: AT (**A, B, D**) and GR (**C**). Scale bar: 2.5 mm.

Medium-sized species (Fig. 3A–D). Wingspan 11.5–15.5 mm (*N* = 99, 47 males, 52 females). Costal margin of forewing white from base to 4/5 of wing length; the rest shining coffee-brown; wing divided into three sections by two pale longitudinal strips bordered by darker shade on both sides; white strip next to fold extends from base towards outer corner but fades before reaching it; median strip runs from 1/5^th^ of the wing’s length from base to outer corner; trailing edge of the forewing narrow, white, barely discernible; colour of area between leading edge and median strip gradually changing from pale brown at base to coffee-brown at wing tip; area between median strip and trailing edge pale yellow; hind wing light brown, with a darker shade at base; abdomen brown, scutellum white, central part of head pale yellow; base of antenna white; flagellum annulated with alternating black and white; hind tibia densely covered with silvery bristles (setae) (Baldizzone 2019).

Male genitalia (Figs 1A, 1B, 2C–K): Gnathos large, broad, rounded, densely spined; basal arm of gnathos rounded; tegumen long and broad, with nearly parallel margins; pedunculus relatively broad, strongly sclerotised; valvula broad, more or less angular in shape; arm of cucullus broad, widening towards to the apex; sacculus elongated, rounded, tapering towards base; vinculum U-shaped. Within phallotheca, cornuti strongly sclerotised and form a loose bundle that spreads out at the apex. Abdominal structures: lack of posterior lateral strut remarkable; proximal edge of transverse strut slightly curved, more sclerotised in middle than elsewhere, distal edge arched, thick; tergal disc (4^th^ tergite) about 3 times longer than wide, covered with about 25–30 small conical spines.

Female genitalia (Fig. 2A, B): Basal plate of papillae anales oval and heavily sclerotised; posterior apophyses relatively short and straight. Anterior apophyses twice as long as segment VIII. Sterigma relatively short and rounded; ostium bursae wide and relatively short, U-shaped. Ductus bursae heavily sclerotised from antrum to spiral loop, covered with rasp-like spicules; dark inner stripe and medial line shorter; proximal ends of ductus bursae narrower and more sclerotised. Corpus bursae ovoid; basal plate of signum slightly sclerotised, C-shaped, posterior part slightly curved and rounded.

##### Etymology.

The specific epithet is derived from the generic name of the host plant, *Chamaecytisus*.

##### Distribution and habitat.

The species has been collected at 11 locations in Hungary and one in Austria so far. The habitat of *C. cytisicolella* in Hungary is moderately dry grassland, primarily meadow steppe and forb-rich fescue-feathergrass steppe on stony hillsides, and loess-covered areas in the hills and lowland areas of the forest steppe region, where *Chamaecytisus austriacus* is abundant. We found cases and imagines in areas where the food plants were growing on loess or sand. The habitat in Austria is a sand steppe on a historically drifting sand dune.

##### Life history, cases and larval development.

Adults fly in April and May, but we caught a male in Hungary on 21 July 2022, and another one on 8 August 2023. This observation does not fit the idea that the species has a single generation. We currently do not know whether these specimens represent a second generation.

The larvae hatch and begin feeding in early September and continue feeding until late October or early November. Larval development is completed during autumn. The final instar overwinters on the host plant, then pupates in the spring without resuming feeding. The length of the pupal stage is unknown.

During 2023, we found 16 larval cases at the loess wall in Kazal-hegy near Lovasberény, Hungary. Twelve larvae were successfully reared on *Chamaecytisus austriacus*, and all developed into imagines. In 2024, we found a total of 112 cases in the settlements listed above.

**Figure 4.**
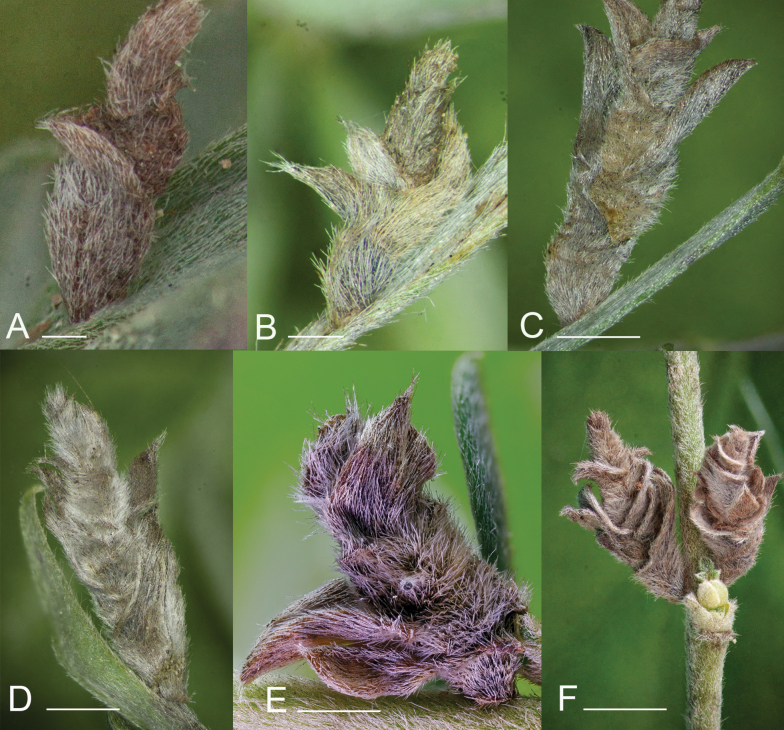
Different stages of *Coleophora cytisicolella* sp. nov. cases on *Chamaecytisus austriacus*. **A**. L1 case in Fejér County, Lovasberény, loess wall, 19 September 2023, leg. AT; **B**. L2 case in Fejér County, Lovasberény, loess wall, 5 October 2023, leg. AT; **C**. L3 case in Fejér County, Lovasberény, loess wall, 5 October 2023, leg. AT; **D**. L5 case in Austria, 3 October 2024, leg. WS.; **E**. L4 case in Fejér County, Lovasberény, loess wall, 25 October 2023, leg. AT; **F**. L5 case in Fejér County, Lovasberény, loess wall, 5 October 2023, leg. AT. Photos: AT (**A, B, C, D, F**) and GR (**E**). Scale of bars: 0.5 mm (**A**); 0.7 mm (**B**); 1.4 mm (**C**); 1.7 mm (**D**); 1.9 mm (**E, F**).

The morphology of the case changes during larval development. The first case is tubular and only 1.5 mm long (Fig. 4A). It is constructed by the small larva in early September, immediately after hatching. The L2 case is 2.5 mm long and is prepared from two pieces of leaf, which are cut out of the leaf tip (Fig. 4B). The case built by the L3 instar is similar in shape and structure to the final case, however smaller in size (Fig. 4C). The L4 case consists of numerous distinct leaf pieces, but its size falls short of that of the final case (Fig. 4D).

The case of the final instar (L5) is a characteristic leaf-case (Fig. 4E, F) (Emmet et al. 1996). It is prepared from leaf pieces by the end of October. At this point, the case is 7.5 mm long but has shrunk to 6 mm after overwintering. The leaf pieces are incorporated into the case approximately half to two-thirds of the length of the case, while the remaining free part is incorporated (Fig. 4C). Each leaf piece is incorporated into the sheath for about half of its length, while the remainder is leafless and smooth. The first protruding leaf pieces are set at 1.5 mm from the mouth. The mouth is perpendicular to the axis of the case. The anal opening is bivalved.

Larval development is completed during autumn. The final instar overwinters on the host plants, then pupates in the spring without resuming feeding. The length of the pupal stage is unknown.

### Molecular results

**Figure 5.**
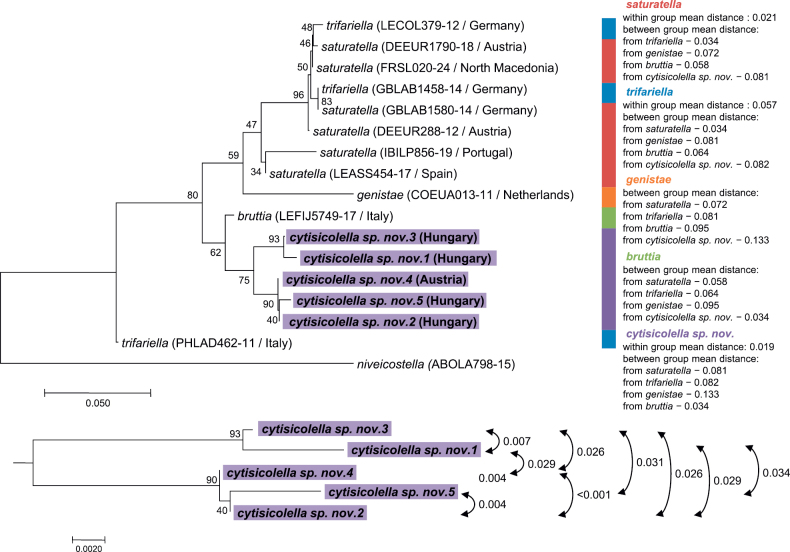
Phylogenetic tree and genetic distances of the examined *Coleophora* haplotypes. The phylogenetic tree was constructed using the ME method implemented in MEGA 7, and node support was estimated with 1000 bootstrap replicates.

Sequencing of the COI barcoding region of the examined *Coleophora cytisicolella* sp. nov. resulted in nucleotide sequences between 573 base pairs (bp) (COLHU031-25) and 644 bp (COLHU025-24). Following alignment with additional haplotype sequences obtained from the BOLD System, the sequences were trimmed to remove sites with missing data from both 5' and 3' ends. Trimming resulted in a 492 bp-long aligned block, which was used in subsequent analyses. The examined sequences of the eight collected *Coleophora cytisicolella* sp. nov. specimens clustered into five haplotypes. Among these haplotypes, haplotype 2 was the most frequent, occurring in four samples, whereas a single specimen represented each of the remaining haplotypes (Table 1). We used these haplotypes for our genetic analysis. Based on the examined 492 bp-long COI barcode fragment, the analysed haplotypes of *C. cytisicolella* sp. nov. formed a monophyletic group, which was a sister group of the haplotype of the Italian *C. bruttia* (Fig. 5). They split into two clusters (BINs: BOLD:AFA2793 and BOLD:AGC5189). The first one consisted of haplotype 1 and 3, whereas the second included haplotype 2, 4, and 5 (Fig. 5). The mean within-group genetic distance was 1.9%, and the distance between the two most distant *C. cytisicolella* haplotypes was 3.4%. Their average distance from the nearest neighbour *C. bruttia* (BOLD: ADI5256) was also 3.4% (Fig. 5).

The mean pairwise genetic distances between C. cytisicolella and the rest of the examined species ranged between 0.081 and 0.133. These values were at least as large as those for the remaining species.

## Discussion

Considering all the evidence presented in this study, we are convinced that *C. cytisicolella* sp. nov. is most likely a distinct species, new to science. We are aware, however, that the analyses of our morphological and genetic data provided incoherent results. The observed morphological differences apparently justify the treatment of *C. cytisicolella* sp. nov. as a distinct species, whereas the evidence from genetic data is weak. Despite the shortcomings of our genetic analyses, we think that the available evidence is still sufficient to treat *C. cytisicolella* sp. nov. as a separate species. In addition to macromorphological characters, such as colouration and wing pattern, genitalia morphology is clearly different between the new species and its closest relative, *C. bruttia*. The magnitude of this difference is comparable to that in other pairs of well-established species in the genus, such as *C. trifariella* and *C. saturatella* (Baldizzone 2019), and *Coleophora uralensis* Toll, 1961 and *Coleophora paradoxella* Toll, 1961 (JT pers. comm.). We are convinced, therefore, that the observed difference between the genitalia of *C. cytisicolella* sp. nov. and *C. bruttia* is sufficiently large to reliably assume either complete reproductive isolation between the two taxa, or at least limited gene flow leading to the emergence of a phylogenetically independent lineage.

Differences in life-history characters also indicate that *C. cytisicolella* sp. nov. has diverged from most species in the C. genistae group. Adults of *C. cytisicolella* sp. nov. fly from late March to late May, whereas *C. trifariella* flies in July, *C. genistae* in July–August, and *C. saturatella* in June–July. The flight period of *C. bruttia* is not known, however. The date of collection (20 May) of the single specimen (Baldizzone 2023) falls within the flight period of *C. cytisicolella* sp. nov., suggesting either little or no divergence in this character or some degree of overlap of the flight periods. However, comparing the flight periods of these species may be inappropriate given climatic differences between the Carpathian Basin and Southern Italy.

Divergence of *C. cytisicolella* sp. nov. from its closest relatives is also demonstrated by our phylogenetic analysis and genetic data. The placement of *C. cytisicolella* sp. nov. as a sister group of *C. bruttia* in the phylogenetic tree is congruent with our data on genitalia morphology. This, along with the mean interspecific genetic distances, suggests that *C. cytisicolella* sp. nov. is more closely related to *C. bruttia* than it is to the rest of the taxa in the *C. genistae* group. The interspecific genetic distances also indicate that *C. cytisicolella* is about as divergent from the examined taxa, except *C. bruttia*, as the rest of the taxa but *C. bruttia* are from one another. This is also congruent with the similarity relations among the genitalia structures. These findings support the notion that *C. cytisicolella* sp. nov. is sufficiently divergent from all but one of its close relatives within the *C. genistae* species group to be separated from them at the specific rank.

However, analyses of our genetic data yielded inconclusive results regarding the taxonomic status of *C. cytisicolella* sp. nov. These results are not suitable to tell whether the observed genetic divergence of *C. cytisicolella* sp. nov. from *C. bruttia* is large enough to separate the two taxa at the specific rank. We found that, on average, *C. cytisicolella* sp. nov. and *C. bruttia* are more divergent than are haplotypes within each of the remaining taxa. We also found, however, that the range of intraspecific genetic distances in *C. cytisicolella* sp. nov. is as large as the mean interspecific genetic distance of *C. cytisicolella* sp. nov. from *C. bruttia*.

It is not likely that the observed intraspecific genetic distances in *C. cytisicolella* sp. nov. are unusually large. Examination of the genitalia in *C. cytisicolella* sp. nov. did not reveal significant differences among specimens, suggesting that individuals with distant haplotypes belong to the same species. Similarly, differentiation owing to geographical isolation is unlikely to account for the relatively large intraspecific genetic distances between *C. cytisicolella* sp. nov. haplotypes, as the geographical origins of the samples (Fig. 5) from the two Pannonian populations (Table 1) are not reflected in their phylogenetic relationships. Instead, these values could result from high within-species genetic diversity of the examined barcoding sequences.

It is possible, however, that interspecific genetic distances are unusually small and thus unreliable. Unfortunately, our estimates may be biased because only one haplotype is available from *C. bruttia*. Without more haplotypes of *C. bruttia*, we cannot obtain better estimates and thus cannot determine the taxonomic status of *C. cytisicolella* sp. nov. on the basis of genetic data alone.

Another possibility is that the small distance values reflect recent divergence between these taxa. Divergence may have resulted in reproductive isolation as suggested by differences in genital morphology (Fig. 3), but has not resulted in deep divergence in the sequenced barcoding region (i.e., incomplete lineage sorting (Maddison 1997)). This seems to be the case in *Coleophora uralensis* Toll, 1961 and *Coleophora paradoxella* Toll, 1961, where the two species are identical in their barcoding sequences but differ sharply in genitalia morphology (JT pers. com.).

The limitations of our study clearly indicate the need for additional work on this group of species. The presented phylogenetic tree of haplotypes suggests that two members of the *C. genistae* group are probably polyphyletic. This may be the result of erroneous assignment of haplotypes to species in the BOLD Database. Also, the taxonomic relationships among members of the *C. genistae* species group, and especially between *C. cytisicolella* sp. nov. and *C. bruttia*, could be better understood if the haplotype sample size in *C. bruttia* were increased along with the amount of data on its ecology and life history.

In this study, we did not address certain questions concerning some aspects of the biology of the new species. For example, members of the C. genistae group are uniformly univoltine (Baldizzone 2019), and there is no evidence that *C. cytisicolella* sp. nov. is different. Nevertheless, two adults of the latter species were collected in July and August, much later than its reported flight period. We do not know whether this indicates the existence of a second, even if partial generation, or is due to some unknown local factors that we did not examine.

Another question is the degree of food-plant specificity of the new species. According to our observations, *C. cytisicolella* sp. nov. is associated only with *Chamaecytisus austriacus* as its larval host plant. We did not find any cases of other *Coleophora* species during surveys of *Chamaecytisus austriacus*. However, we did not survey other, closely related plant species for the presence of *C. cytisicolella* sp. nov. As a consequence, we still do not know whether this species is monophagous, specializing solely on *Chamaecytisus austriacus*, or whether it can develop on multiple plant species. To obtain additional information on food-plant specialization, it would be essential to increase sampling efforts in habitats where *Chamaecytisus austriacus* and other potential food plants occur. This could result not only in the discovery of new populations but would also contribute to a better understanding of the geographical distribution of this new *Coleophora* species.

## Supplementary Material

XML Treatment for Coleophora cytisicolella

## Additional information

### Use of AI

No use of AI was reported.

### Author ORCIDs

Attila Sándor Takács https://orcid.org/0000-0003-3427-538X

Wolfgang Stark https://orcid.org/0009-0005-5623-1259

Csaba Szabóky https://orcid.org/0000-0002-1290-0976

Miklós Bozsó https://orcid.org/0000-0003-1285-636X

Klaudia Kőszegi https://orcid.org/0009-0009-3825-6507

Gábor Lendvai https://orcid.org/0000-0001-9301-701X

Ignác Richter https://orcid.org/0000-0001-8122-8709

Gábor Sramkó https://orcid.org/0000-0001-8588-6362

Sándor Jordán https://orcid.org/0000-0002-3556-4127

### Data availability

All of the data that support the findings of this study are available in the main text.
